# Seroprevalence of Infections with Dengue, Rift Valley Fever and Chikungunya Viruses in Kenya, 2007

**DOI:** 10.1371/journal.pone.0132645

**Published:** 2015-07-15

**Authors:** Caroline Ochieng, Petronella Ahenda, Amy Y. Vittor, Raymond Nyoka, Stella Gikunju, Cyrus Wachira, Lilian Waiboci, Mamo Umuro, Andrea A. Kim, Leonard Nderitu, Bonventure Juma, Joel M. Montgomery, Robert F. Breiman, Barry Fields

**Affiliations:** 1 Center for Global Health Research, Kenya Medical Research Institute, Nairobi, Kenya; 2 Global Disease Detection Program, United States Center for Disease Control and Prevention, Nairobi, Kenya; 3 Division of Infectious Diseases and Global Medicine, Emerging Pathogens Institute, University of Florida, Gainesville, Florida, United States of America; 4 Ministry of Public Health and Sanitation, Nairobi, Kenya; 5 Emory University, Atlanta, Georgia, United States of America; 6 University of Nairobi, Nairobi, Kenya; 7 Africa Biomedical Laboratories, Nairobi, Kenya; George Mason University, UNITED STATES

## Abstract

Arthropod-borne viruses are a major constituent of emerging infectious diseases worldwide, but limited data are available on the prevalence, distribution, and risk factors for transmission in Kenya and East Africa. In this study, we used 1,091 HIV-negative blood specimens from the 2007 Kenya AIDS Indicator Survey (KAIS 2007) to test for the presence of IgG antibodies to dengue virus (DENV), chikungunya virus (CHIKV) and Rift Valley fever virus (RVFV).The KAIS 2007 was a national population-based survey conducted by the Government of Kenya to provide comprehensive information needed to address the HIV/AIDS epidemic. Antibody testing for arboviruses was performed on stored blood specimens from KAIS 2007 through a two-step sandwich IgG ELISA using either commercially available kits or CDC-developed assays. Out of the 1,091 samples tested, 210 (19.2%) were positive for IgG antibodies against at least one of the three arboviruses. DENV was the most common of the three viruses tested (12.5% positive), followed by RVFV and CHIKV (4.5% and 0.97%, respectively). For DENV and RVFV, the participant’s province of residence was significantly associated (P≤.01) with seropositivity. Seroprevalence of DENV and RVFV increased with age, while there was no correlation between province of residence/age and seropositivity for CHIKV. Females had twelve times higher odds of exposure to CHIK as opposed to DENV and RVFV where both males and females had the same odds of exposure. Lack of education was significantly associated with a higher odds of previous infection with either DENV or RVFV (p <0.01). These data show that a number of people are at risk of arbovirus infections depending on their geographic location in Kenya and transmission of these pathogens is greater than previously appreciated. This poses a public health risk, especially for DENV.

## Introduction

Arthropod-borne viruses (arboviruses) are classified into three main families; Togaviridae (e.g., chikungunya virus (CHIK)), Flaviviridae (e.g., dengue virus (DENV)) and the Bunyaviridae (e.g., Rift Valley Fever virus (RVFV)). They cause a range of clinical syndromes in humans ranging from a self-limiting, febrile illness to life-threatening encephalitis or hemorrhagic fever. Arboviruses are a major cause of morbidity in sub-Saharan Africa [1]; however, most infections caused by arboviruses remain undiagnosed or misdiagnosed as malaria, typhoid, dysentery, or bacterial meningitis due to the nonspecific nature of the clinical signs and the lack of readily available laboratory testing [2–4][2–4]. Hence, the incidence of arboviral diseases and the public health burden are likely greatly underestimated. The World Health Organization (WHO) estimates that DENV causes more than 50 million clinical cases globally each year [5]. In the United States, eastern equine encephalitis virus (family *Togaviridae*, genus *Alphavirus*), St. Louis encephalitis virus and West Nile virus (both in the family *Flaviviridae*, genus *Flavivirus*) are important causes of viral encephalitis.

In East Africa, multiple arbovirus epidemics have occurred in the past two decades. In 1982, the first outbreak of dengue was reported along the Kenyan Coast [6]. Approximately 30 years later, in 2011, a dengue outbreak occurred in North Eastern Kenya [7]. Dengue virus activity continued in this region, and in early 2013 a dengue outbreak was detected in Mombasa, Kenyan Coast [7]. These recent dengue outbreaks involved three different dengue serotypes (DEN-1, DEN-2 and DEN-3). Dengue appears to occur as an endemic disease in Kenya with periodic outbreaks primarily occurring along the coast. DENV infection causes a spectrum of symptoms, from mild, non-specific symptoms to classic dengue fever, with high fevers and severe arthralgia. Reinfection with a new serotype can lead to dengue hemorrhagic fever or dengue shock syndrome, which is a severe form of dengue fever.

CHIKV infection is endemic in Africa and Asia, where numerous outbreaks have been reported since the 1950s.There are three genotypes of CHIKV, which are the West African, Central/East African (CEA) and Asian genotypes [8]. Since 2005, CHIKV of the CEA genotype has spread worldwide from initial outbreaks on Lamu in 2004 [9], Mombasa in 2004 [10], Comorros Island in 2005 [11] and in India affecting millions [8]. The onset of CHIK epidemics is rapid, often with high attack rates as seen in the 2005–2006 on La Réunion outbreak [12]. Concurrent epidemics of DEN and CHIK have been reported from Asia [13] and Africa [14]. In 2006, a combined outbreak of dengue fever and chikungunya fever occurred near the Madagascan city. The coastal region of Kenya was shown to be greatly affected in the recent outbreak of CHIK fever [15, 9].There have also been sporadic imported cases and occasional outbreaks of CHIK fever in other regions outside Africa and Asia such as Italy in 2007 [16] and in France in 2010 [17, 18]. In late 2013, autochthonous transmission of the Asian genotype of CHIKV was reported for the first time in the Caribbean. By December 2014, over one million suspected cases were reported from this region, with local transmission established in 41 countries or territories in the Americas (CDC: http://www.cdc.gov/chikungunya/geo/americas.html). This latest outbreak in the Americas underscores the importance of CHIKV infection as a global health threat.

In 1997–98, an extensive outbreak of Rift Valley fever (RVF) virus occurred in East Africa. Ten years later, another outbreak of RVF virus occurred affecting Kenya, Tanzania and Somalia [19–21]. In Kenya alone, over 150 human lives and countless animals were lost in this outbreak [21], posing a great economic burden on the region. In Kenya, there are very scarce data on seroprevalence of DEN, CHIK and RVF. Previous studies [22, 23, 24] on arbovirus seroprevalence in Kenya only covered selected regions in the country. In this study, a subset of samples from the first Kenya AIDS Indicator Survey (KAIS 2007), a national population-based household survey of persons aged 15–64 years, was used to test for Immunoglobulin G (IgG) antibodies against Rift Valley Fever virus, Chikungunya virus and Dengue virus. This was to assess the magnitude and geographic distribution of exposure to these arboviruses and the risk factors associated with these arboviral agents. This study also sought to identify the populations most-at-risk of infection and acute outbreaks due to these arboviruses in order to target public health interventions.

## Materials and Methods

### Study Design

Specimens were collected through the KAIS 2007, described in detail elsewhere [25]. This study was a population-based cross-sectional house hold survey of persons aged 15–64 years with an aim to provide representative information on the status of HIV/AIDS in Kenya. The survey used a stratified, two stage cluster sample, designed to generate representative estimates of HIV prevalence at the national and provincial level. The first stage of sampling involved selecting 415 clusters from a national sampling frame, the National Sample Survey and Evaluation Programme IV (NASSEP IV), and the second stage involved a selection of households per cluster with equal probability of selection in the rural‐urban strata within each district country-wide. Household members were interviewed to collect information on demographics, behaviors, and access to services. Blood specimens were collected to test for HIV infection. Remnant specimens from respondents who consented to future unspecified testing of their blood samples were stored at -80°C. None of the stored HIV-positive samples were available at the time of arbovirus testing. Therefore, a sub-sample of HIV-negative samples was randomly selected to determine IgG antibody titers to RVFV, CHIKV and DENV. Sample sizes necessary to establish representative seroprevalence by province were calculated for each of the three arboviral infections, taking into account the design effect and intracluster correlation (ρ, assumed to be 0.2) with a precision of 0.025 [26].

### Laboratory

Sera from the study subjects were tested by enzyme immunoassays for IgG antibody to DEN, CHIK and RVF viruses. Briefly, DENV IgG was detected using a “two-step” sandwich-type immunoassay. This was done according to the manufacturer’s instructions using the InBios DENV DetectIgG ELISA kit. The DENV IgG negative control which represents a non-reactive serum, DENV IgG positive and the test serum samples were first diluted with sample dilution buffer for DENV IgG. Thereafter, these were incubated in micro-titre plates pre-coated by the manufacturer with monoclonal antibody bound to recombinant DENV antigen. After subsequent incubation and washing, the wells were treated with specific conjugate and thereafter with substrate. A stop solution was then added and the absorbance measurement read at 450nm using the ELx800 absorbance microplate reader to determine the optical density.

CHIK and RVF virus detection required coating of the plates prior to the assay with their respective specific antigens (CDC Atlanta- Special Pathogens Laboratory). This was followed by overnight incubation and washing before addition of the test samples. After subsequent incubation and washing, the wells were treated with specific conjugates respectively for each of the two immunoassays. Thereafter, the wells were incubated with a substrate for which the enzymatic turnover was determined by measuring the optical density using the ELx800 absorbance reader. For each assay, a set of positive and negative controls was included.

### Data Analysis

All analyses were conducted using survey procedures in Statistical Analysis Software (SAS) version 9.3 (SAS Institute Inc, Cary North Carolina, USA). The calculation of seroprevalence was adjusted to account for the variation in sampling of the study population. Sampling weights for households, individual interviews, blood draws and pathogen testing were used to correct for unequal probability of selection and to adjust for non-response to the questions asked during the survey [26]. These were used to represent the larger population from which the sample was drawn. A cluster was required to have a minimum of 3 samples on sampling and when this was not achieved due to the small sample size of negative HIV blood samples, non-positive sampling weights were obtained and were therefore excluded from analysis. Frequency distributions were calculated for the three arboviruses. Bivariate and multivariate logistic regression models were used to test the association between the outcome variables with sex, age, education level, wealth and province. In bivariate analysis, variables that were associated with the outcome at a significance level of p-value < 0.05 were considered to be statistically significant, while those with p-value < 0.1 were considered in the multivariate logistic regression model. This is helpful in identifying variables that, by themselves, are not significantly related to the outcome but make an important contribution in the presence of other variables. Traditional levels such as 0.05 can fail to identify variables known to be important [27–28] and that’s why any variable that was significant at the 0.1 level was put in the model.

### Ethical Statement

This study was approved by the Scientific Steering Committee (SSC) and the Ethical Review Committee at the Kenya Medical Research Institute (KEMRI) (SSC # 1209) and by the Institutional Review Board (IRB) at the U.S. Centers for Disease Control and Prevention (CDC) (IRB Protocol # 5169). The analysis was conducted on a sub-sample of stored serum specimens from survey participants who consented to storage of specimens for future testing of other infectious diseases. All the specimens that were used for this analysis were unlinked from personal identifiers. There was no contact with participants and no new data collection was required for the purpose of this activity.

## Results

A total of 17,970 persons participated in the survey, of whom 15,853 (88.4%) provided blood samples for HIV testing. Of these, 14,984 (94.5%) serum samples were stored and available for future testing, including 943 HIV-positive and 14,041 HIV-negative serum samples. Of the HIV-negative serum samples available, we randomly selected 1,091 (7.8%) specimens for testing. All eight provinces were represented in this study to ensure an equal distribution of study subjects. Out of the 1,091 participants who provided blood samples which were tested, half (50.8%) were aged 15–29 years. There were more females sampled as compared to males in all categories (Table 1). All socioeconomic classes were represented with the lowest and highest quintiles of wealth being the most sampled groups. Over half of the participants had completed at least their primary and secondary education (55.5%). There was a higher number of KAIS participants whose samples were tested for the current study from Rift Valley Province (19.6%) compared to other provinces of Kenya (Table 1).

**Table 1:** Demographic characteristics of study participants aged 15–64 years who were sampled for serologic survey of DEN, RVF and CHIK viruses, Kenya 2007.

Demographic Characteristic	Category	Total N (%;95% CI)	Male n (%; 95% CI)	Female n (%; 95% CI)
Age category	15–29	529 50.8 (46.4–55.2)	208 51.6 (45.6–57.6)	321 50.2 (44.4–56.1)
	30–49	395 34.9 (31.2–38.7)	158 34.5 (28.6–40.4)	237 35.2 (30.1–40.3)
	50–64	167 14.3 (11.7–16.9)	64 13.8 (9.8–17.8)	103 14.6 (10.7–18.4)
Education	No primary	223 15 (12.4–17.6)	59 9.9 (6.6–13.2)	164 18.1 (14.8–21.5)
	Incomplete primary	295 29.5 (25.8–33.1)	116 28.3 (22.7–33.8)	179 30.2 (25.4–34.9)
	Complete primary & secondary +	573 55.5 (51.7–59.4)	255 61.8 (56.4–67.3)	318 51.7 (46.8–56.6)
Wealth Quintiles	Lowest	234 16.2 (13.3–19)	89 17 (12.6–21.3)	145 15.7 (12.4–19.1)
	Second	189 17.3 (14.3–20.3)	80 19.3 (14.4–24.2)	109 16 (12.2–19.8)
	Middle	188 17.6 (14.9–20.4)	71 17.1 (13.1–21.1)	117 17.9 (14.5–21.3)
	Fourth	186 19.5 (16.5–22.4)	69 19.4 (13.9–24.9)	117 19.5 (15.9–23.2)
	Highest	294 29.5 (25.6–33.3)	121 27.2 (22–32.4)	173 30.8 (25.2–36.4)
Province	Nairobi	153 12.9 (11–14.7)	65 13.6 (10–17.2)	88 12.4 (9–15.8)
	Central	150 14.6 (12.8–16.3)	59 14 (10.5–17.5)	91 14.9 (11.9–17.9)
	Coast	129 8.3 (7–9.6)	55 10 (7.6–12.5)	74 7.3 (5.2–9.4)
	Eastern	112 11.5 (10.1–13)	37 9.6 (6.9–12.3)	75 12.7 (10.1–15.4)
	North Eastern	140 5.9 (4.1–7.8)	55 6.9 (4.1–9.8)	85 5.3 (3.8–6.9)
	Nyanza	154 15.2 (13.2–17.3)	62 14.6 (11.2–18)	92 15.6 (12.4–18.8)
	Rift Valley	117 19.6 (16.1–23.1)	45 18.6 (13.3–23.9)	72 20.1 (15.2–25)
	Western	136 12 (10.6–13.3)	52 12.6 (9.3–15.8)	84 11.6 (9.4–13.7)

CI- is the confidence interval

+ = and above

N, n = number of persons

**Fig 1:**
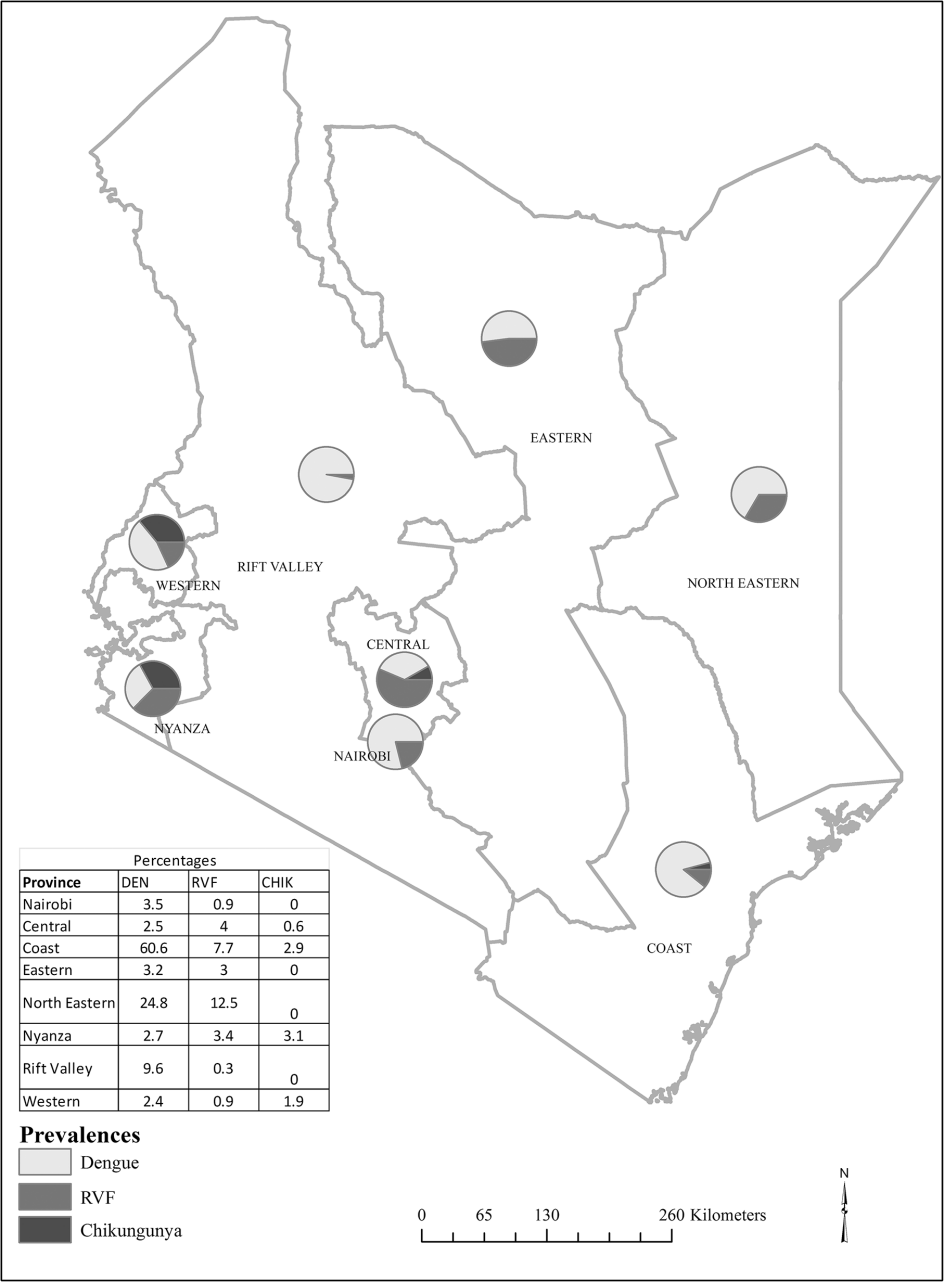
Seroprevalence of dengue, Rift Valley fever and chikungunya virus infections in Kenya.

Overall, the seroprevalence of dengue was 12.5% (95% CI 8.7–16.3) in Kenya. Seropositivity varied by province, with the higher seroprevalence in Coast province, at 60.6% (95% CI 51.2–70.1) and the lowest was in Western province, at 2.4% (95% CI 0.0–4.8) (Fig 1). In bivariate analysis (Table 2), the odds of dengue seropositivity varied significantly by education level (p<0.01), province of residence (p<0.01), and wealth index (p = 0.04). In addition, persons aged 50–69 years had three times higher odds of dengue seropositivity than persons aged 15–29 years (p = 0.02). After controlling for demographic factors, province of residence remained independently and significantly associated with DENV infection. Compared to North Eastern province, higher adjusted odds of dengue seropositivity was found in Coast province (OR 4.9; 95% CI 2.2–11.0) while lower adjusted odds of dengue seropositivity were found in Nairobi (OR 0.1; 95% CI 0.01–0.4), Central (OR 0.1; 95% CI 0.02–0.3), Eastern (OR 0.1; 0.03–0.4), Nyanza (OR 0.1; 95% CI 0.03–0.3), and Western (OR 0.1; 95% CI 0.03–0.3) provinces.

**Table 2:** Prevalence and factors associated with dengue virus among persons aged 15–64 years, Kenya, 2007.

Variable	Total (N)	Total—Pos (n)	Total—Pos % (95% confidence interval)	Unadjusted odds ratio (95% confidence interval)	p_value	Adjusted odds ratio (95% confidence interval)	M_p_value
**Sex**							
Male	430	57	12.3 (8.0–16.5)	REF			
Female	661	86	12.6 (7.9–17.4)	1.03 (0.65–1.64)	0.89		
**Age category (years)**							
15–29	529	44	8.7 (4.1–13.2)	REF	0.06		
30–49	395	62	13.3 (9.3–17.4)	1.62 (0.84–3.12)		1.51 (0.74–3.10)	0.26
50–64	167	37	23.9 (9.4–38.3)	3.3 (1.22–8.9)		3.06 (0.99–9.52)	0.05
**Education level**							
No primary	223	55	25.8 (16.4–35.2)	3.44 (1.47–8.06)	< .01	1.20 (0.43–3.38)	0.73
Incomplete primary	295	26	6.5 (3.8–9.2)	0.69 (0.34–1.4)		0.54 (0.22–1.35)	0.19
Complete primary & secondary +	573	62	9.2 (4.5–13.8)	REF			
**Wealth Quintiles**							
Lowest	234	44	18.4 (11.2–25.7)	1.87 (0.82–4.25)	0.04	0.50 (0.20–1.26)	0.14
Second	189	17	11.6 (4.0–19.2)	1.08 (0.41–2.89)		0.46 (0.16–1.34)	0.16
Middle	188	21	9.4 (4.9–14.0)	0.86 (0.36–2.06)		0.44 (0.16–1.22)	0.11
Fourth	186	26	11.5 (6.3–16.7)	1.07 (0.48–2.41)		0.72 (0.24–2.15)	0.56
Highest	294	35	10.8 (4.2–17.4)	REF			
**Province**							
Nairobi	153	8	3.5 (0.5–6.4)	0.11 (0.04–0.29)	< .01	0.07 (0.01–0.37)	< .01
Central	150	4	2.5 (0.0–5.2)	0.08 (0.03–0.24)		0.06 (0.02–0.25)	< .01
Coast	129	78	60.6 (51.2–70.1)	4.66 (2.68–8.13)		4.92 (2.19–11.04)	< .01
Eastern	112	6	3.2 (0.3–6.1)	0.1 (0.04–0.28)		0.11 (0.03–0.37)	< .01
North Eastern	140	32	24.8 (17.5–32.1)	REF			
Nyanza	154	5	2.7 (0.3–5.0)	0.08 (0.03–0.22)		0.10 (0.03–0.33)	< .01
Rift Valley	117	5	9.6 (0.0–22.0)	0.32 (0.07–1.41)		0.29 (0.10–0.80)	0.02
Western	136	5	2.4 (0.0–4.8)	0.07 (0.02–0.23)		0.10 (0.03–0.33)	< .01

cut off point to include factors in multivariate is pvalue <0.1

p value is the global p value for the bivariate variable

M_OR_CI is the multivariate odds ratio

M_p_value is the category multivariate p value

REF- reference

+ = and above

N, n = number of persons

The national seropositivity of antibody to Rift Valley Fever (RVF) virus was 4.5% (95% CI 2.7–6.3). The highest seropositivity of RVF virus infection was in North Eastern (12.5%, 95% CI 0.5–24.5) and Coast provinces (7.7%, 95% CI 0.5–14.8) and the lowest seropositivity was in Rift Valley province (0.3%, 95% CI 0.0–0.9) (Fig 1). The odds of RVF seropositivity was significantly high in Central (p = 0.02), Coast (p<0.01), North Eastern (p<0.01), and Nyanza (p = 0.04) provinces (Table 3). In addition, individuals from the lowest wealth quintile had higher odds of RVF seropositivity than those in the highest wealth quintile (Table 3). The elderly (50–64 years) had higher odds of exposure to RVF than the younger group (15–29 years). Lack of education was significantly associated with RVF seropositivity (p <0.01) by bivariate analysis. In multivariate analysis, the association between dengue seropositivity and age, education level, and wealth index fell out of significance. However, province of residence remained independently and significantly associated with dengue seropositivity. Compared to Rift Valley province, the odds of dengue infection was 20 times higher in Central (OR 20.0; 95% CI 2.4–167.9), 30 times higher in Coast (OR 30.3; 95% CI 3.1, 292.2), 28 times higher in North Eastern (OR 27.6; 95% CI 2.7–282.5), and 12 times higher in Nyanza (OR 11.7; 95% CI 1.2–113.7).

**Table 3:** Prevalence and factors associated with RVF virus among persons aged 15–64 years, Kenya, 2007.

Variable	Total (N)	Total—Pos (n)	Total—Pos % (95% confidence interval)	Unadjusted odds ratio (95% confidence interval)	p_ value	Adjusted odds ratio (95% confidence interval)	M_p_value
**Sex**							
Male	419	26	5.4 (2.4–8.5)	ref			
Female	638	31	3.7 (2.2–5.3)	0.67 (0.40–1.15)	0.15		
**Age category (years)**	** **	** **	** **	** **		** **	** **
15–29	516	19	3.3 (1.6–5.1)	ref	0.05		
30–49	378	20	4.2 (1.1–7.4)	1.28 (0.57–2.86)		0.83 (0.32–2.11)	0.69
50–64	163	18	8.7 (3.1–14.2)	2.76 (1.22–6.26)		2.25 (0.90–5.62)	0.08
**Education level**							
No primary	211	31	10.0 (3.5–16.4)	4.69 (2.05–10.72)	< .01	0.92 (0.48–1.79)	0.81
Incomplete primary	283	7	3.4 (0.3–6.4)	1.48 (0.52–4.20)		0.97 (0.31–3.05)	0.96
Complete primary & secondary +	563	19	2.3 (1.1–3.5)	ref			
**Wealth Quintiles**							
Lowest	223	32	11.1 (5.3–16.9)	7.81 (2.25–27.13)	< .01	4.22 (0.72–24.64)	0.11
Second	183	7	4.4 (0.0–8.9)	2.87 (0.61–13.42)		1.68 (0.23–12.34)	0.61
Middle	184	6	2.7 (0.4–5.1)	1.77 (0.44–7.11)		1.18 (0.19–7.22)	0.85
Fourth	181	5	2.2 (0.1–4.3)	1.40 (0.32–6.06)		0.93 (0.12–6.89)	0.94
Highest	286	7	1.6 (0.0–3.3)	ref			
**Province**							
Nairobi	151	2	0.9 (0.0–2.5)	3.16 (0.23–43.67)	< .01	5.37 (0.27–105.41)	0.27
Central	144	6	4.0 (0.8–7.3)	14.12 (1.61–124.05)		20.03 (2.39–167.89)	0.01
Coast	125	11	7.7 (0.5–14.8)	28.08 (2.99–264.19)		30.26 (3.13–292.15)	< .01
Eastern	107	3	3.0 (0.0–6.5)	10.29 (0.99–107.04)		10.75 (1.02–113.18)	0.05
North Eastern	131	28	12.5 (0.5–24.5)	48.10 (4.91–471.49)		27.60 (2.70–282.50)	0.01
Nyanza	151	4	3.4 (0.0–7.3)	11.75 (1.14–120.90)		11.68 (1.20–113.71)	0.03
Rift Valley	112	1	0.3 (0.0–0.9)	ref			
Western	136	2	0.9 (0.0–2.2)	3.17 (0.28–36.12)		2.72 (0.23–32.69)	0.43

cut off point to include factors in multivariate is pvalue <0.1

p value is the global p value for the bivariate variable

M_OR_CI is the multivariate odds ratio

M_p_value is the category multivariate p value

Ref- reference

+—and above

N, n = number of persons

The overall national CHIKV seropositivity was approximately 1% (95% CI 0.2–1.7). The seropositivity was high from individuals from the Coast and Nyanza Provinces as shown in Fig 1.The highest prevalence was in Nyanza (3.1%; 95% CI 0.0–7.9) followed by Coast province (2.9; 95% CI 0.0–6.8) while the lowest seropositivity was in Central province (0.6%; 0.0–1.7). Females were 12 times more likely to have CHIK than the males (p = 0.02) (Table 4). There was no significant association of CHIKV with age, province, education level or socioeconomic status.

**Table 4:** Prevalence and factors associated with chikungunya virus among persons aged 15–64 years, Kenya, 2007.

Variable	Total (N)	Total-Pos (n)	Total—Pos % (95% confidence interval)	Unadjusted odds ratio (95% confidence interval)	p_value	Adjusted odds ratio (95% confidence interval)	M_p_value
**Sex**	** **	** **	** **	** **	** **	** **	** **
Male	355	1	0.1 (0.0–0.4)	ref			
Female	554	9	1.5 (0.3–2.6)	11.54 (1.40–95.38)	0.02	12.09 (1.46–99.86)	0.02
**Age** (**years**)							
15–29	452	8	1.5 (0.3–2.6)	3.37 (0.80–14.15)	0.1	3.57 (0.84–15.21)	0.09
30–49	457	2	0.4 (0.0–1.1)	ref			
**Education level**							
No primary & incomplete primary	433	8	1.6 (0.2–3.1)	4.38 (0.75–25.49)	0.1		
Complete primary & secondary +	476	2	0.4 (0.0–0.9)	ref			
**Wealth Quintiles**							
Lowest	194	2	0.7 (0.0–1.9)	1.87 (0.20–17.13)	0.47		
Second	164	2	1.3 (0.0–3.1)	3.37 (0.40–28.07)			
Middle	158	2	0.9 (0.0–2.3)	2.41 (0.31–18.96)			
Fourth	154	2	2.3 (0.0–5.5)	5.98 (0.77–46.60)			
Highest	239	2	0.4 (0.0–0.9)	ref			
**Province**							
Central	121	1	0.6 (0.0–1.7)	ref	0.52		
Coast	108	2	2.9 (0.0–7.9)	5.06 (0.35–72.76)			
Nyanza	134	4	3.1 (0.0–6.8)	5.51 (0.54–56.56)			
Western	122	3	1.9 (0.0–4.0)	3.21 (0.32–32.09)			

cut off point to include factors in multivariate is pvalue <0.1

p value is the global p value for the bivariate variable

M_OR_CI is the multivariate odds ratio

M_p_value is the category multivariate p value

Ref- reference

+—and above

N, n = number of persons

## Discussion

While arboviruses are considered a major public health problem in tropical countries, most infections go undetected or misdiagnosed [24]. Arboviral infections are non-specific in their clinical presentations and are often not easily clinically distinguishable from other commonly occurring infections in tropical settings, such as malaria and typhoid, and most clinical facilities are not equipped with rapid diagnostic tests for these pathogens. The findings of this study suggest that during a lifetime exposure to arboviruses like DENV, CHIKV and RVF virus is not unusual in Kenya. Bivariate analysis of factors associated with anti-DENV seropositivity demonstrated an association between exposure to DENV with the participant’s province of residence, age and literacy level. Prevalence of antibodies to DENV was highest in the Coast followed by North Eastern province. This could be due to the abundance of *Aedes* mosquito vectors that transmit this virus in these regions [29]. The previous outbreak of dengue (1982 outbreak) in the Coast [6] could also have contributed to high seroprevalence, since older adults (50–64 years) showed the highest seropositivity to DENV IgG antibodies. The location of the Mombasa port which facilitates shipping traffic between Asia (which experiences Dengue outbreaks) and E. Africa may also be an important contributing factor to the high seroprevalence observed in the region. The frequent movement of people between N. Eastern province and Somalia (where dengue outbreaks have occurred) may also contribute to the high seroprevalence of Dengue in N. Eastern province. There was also a significant association between DENV seropositivity and lack of formal education. One of the predisposing factors might be a high frequency of outdoor activities because many residents in the area are pastoralists, often with limited formal education, who are highly mobile as they move with their livestock for grazing. These activities conceivably increase exposure to day feeding *Ae*. *aegypti* mosquitoes, the principal vectors of DENV. The movement of pastoralists between the Coast province and N. Eastern province may also have contributed to the high seropositivity to DENV IgG antibodies in these two provinces. However, multivariate analysis showed that there was no significant association between DENV exposure and education level. There was also no association between DENV and the socioeconomic status of the population that was sampled in this study by both bivariate and multivariate model. Thus, when controlling for geography, education and socioeconomic status are not associated with seroprevalence of dengue infection. In addition both males and females stood equal chances of contracting dengue virus infection.

RVFV was most prevalent in North Eastern and Coast provinces in Kenya. In 2006/2007, Kenya experienced a major RVFV outbreak affecting North Eastern and Coastal provinces [19]. There is also abundance of the RVF mosquito vectors in these regions thus acting as a predisposing factor to the population that resides here [20–21]. Animal slaughter is common in these regions—slaughtering infected livestock has been shown to be strongly associated with RVFV infection [20]. The intense practice of pastoralism by the N. Eastern people and movement of animals between N. eastern and Coast provinces are also possible factors that could have led to exposure to RVF infections. The prevailing climatic conditions such as high temperatures in the two provinces and occasional flooding also favour the emergence and survival of RVF infected mosquitoes in the areas. We demonstrated a significant association of RVF seropositivity with age, level of formal education, geographical location and socioeconomic status of the population under study by bivariate analysis. The odds of RVF seropositivity in the elderly was twice that of the younger age group and those who had no education background were also the most likely to be seropositive. On the other hand, multivariate analysis showed that age, level of education and socioeconomic status had no significant relationship with RVF seropositivity. Seropositivity for RVFV IgG was significantly associated with geographic regions which had experienced outbreaks previously on bivariate and multivariate analyses (P≤.05).

IgG antibodies to CHIKV were found in people from the Coastal and Nyanza provinces of Kenya. The Coast region experienced an outbreak of CHIKV in 2004 which would have exposed the population to the virus [9], and CHIKV is also transmitted by *Aedes* mosquitoes which might explain some overlap with geographic distribution of DENV exposure. *Ae*. *aegypti* mosquitoes have adapted to more urban domestic habitats, they have exploited a wide range of artificial containers such as vases, water tanks and tires that are often associated with human habitations [30]. The vectors also prefer mammalian hosts [31] and will preferentially feed on humans, even in the presence of alternative hosts [32]. The presence of IgG antibodies to CHIKV from individuals from Nyanza likely indicates circulation of the virus after the 1970 outbreak [33]. These findings confirm reports by Sutherland and others [24]. Overall, there was limited CHIKV seropositivity in Kenya compared to the other two arboviruses. Given the low CHIK seroprevalence, the study may have been underpowered to detect significant associations. Since DENV and CHIKV utilize the same vectors, the risk factors for disease acquisition should be the same, but we could not demonstrate this. The North Eastern and Coast provinces of Kenya seemed to be most affected by arboviruses. This confirms the findings of an earlier vector survey [34] that most arboviruses were isolated from mosquitoes from the N. Eastern province of Kenya. This was associated with the abundance of mosquito vectors and the environmental conditions in the region which support their survival. The principal vector for DEN and CHIK viruses (*Ae*. *aegypti*) is abundant in the Coast province [29] and this may also explain the IgG seropositivity to these arboviruses in this region.

This study has certain limitations. There was limited sample volume even after dilution of available specimens. This prevented confirmatory testing by plaque reduction neutralization test (PRNT) for these arboviruses to rule out cross reactivity. Therefore, the data may indicate cross reactivity especially for flaviviruses and alphaviruses. In this paper, we report CHIK seropositivity of 1% as opposed to 34% that was reported from a serosurvey in Kenya by Mease et al. [22]. The reasons for this discrepancy could be due to the differences in the sampling sites and the method of sampling. In the Mease et al study, sampling was conducted in three districts while in the current study, sampling occurred in all the eight provinces in Kenya. The former study also included individuals regardless of their HIV status while in the later, only a subset of HIV negative samples were used due to unavailability of HIV positive samples. The younger age groups (below 15 years) were also not represented in this study and yet they also play an important role in the epidemiology of the three arbovirus infections. The information generated from this study has identified areas of disease foci in Kenya that require further investigation to determine the burden of disease through active surveillance and might suggest geographically and risk targeted strategies for prevention and reducing the burden of arbovirus-associated diseases.

## Conclusion

The findings of this study suggest that Kenyans, especially in certain geographic locations, are highly likely to be exposed to arboviruses over the course of their lifetime. These arboviral diseases may represent significant public health concerns in Kenya, worthy of further study and potentially of more active interventions to prevent and control these infections.
